# Safety Profile of Antipsychotics as Predictors of the Quality of Life in Patients with Schizophrenia—An Inpatient Welfare Institution-Based Cross-Sectional Study

**DOI:** 10.3390/ph18060777

**Published:** 2025-05-23

**Authors:** Aleksandra D. Petrovic, Ana M. Barjaktarevic, Olivera Z. Kostic, Sara S. Mijailovic, Slobodan M. Jankovic, Marija V. Andjelkovic, Marijana S. Stanojevic Pirkovic, Katarina D. Parezanovic Ilic, Vladimir S. Janjic, Jana Mojsilovic, Jana Arsenijevic, Danijela B. Jovanovic, Sanja Knezevic, Nevena Folic, Milovan Stevic, Dejana Ruzic Zecevic, Nemanja Z. Petrovic, Marina J. Kostic

**Affiliations:** 1 Department of Pharmacology and Toxicology, Faculty of Medical Sciences, University of Kragujevac, 34000 Kragujevac, Serbia; aleksandra-ph@hotmail.com (A.D.P.); slobnera@gmail.com (S.M.J.); dejana.zecevic@gmail.com (D.R.Z.); dr.nemanja.z.petrovic@gmail.com (N.Z.P.); marrina2006kg@yahoo.com (M.J.K.); 2 Department of Pharmacy, Faculty of Medical Sciences, University of Kragujevac, 34000 Kragujevac, Serbia; ana.radovanovickg@gmail.com (A.M.B.); olivera.milovanovic09@gmail.com (O.Z.K.); 3 Center for Harm Reduction of Biological and Chemical Hazards, Faculty of Medical Sciences, University of Kragujevac, 34000 Kragujevac, Serbia; marijabcd@gmail.com (M.V.A.); vladadok@yahoo.com (V.S.J.); 4 Department of Medical Statistics and Informatics, Faculty of Medical Sciences, University of Kragujevac, 34000 Kragujevac, Serbia; saramijailovic212@gmail.com; 5 Department of Medical Biochemistry, Faculty of Medical Sciences, University of Kragujevac, 34000 Kragujevac, Serbia; 6 Department of Physical Medicine and Rehabilitation, Faculty of Medical Sciences, University of Kragujevac, 34000 Kragujevac, Serbia; katarinaparezanovicilic@gmail.com; 7 Department of Psychiatry, Faculty of Medical Sciences, University of Kragujevac, 34000 Kragujevac, Serbia; 8 Department of Dentistry, Faculty of Medical Sciences, University of Kragujevac, 34000 Kragujevac, Serbia; milovan_stevic@yahoo.com; 9 Department of Physiology, Faculty of Medical Sciences, University of Kragujevac, 34000 Kragujevac, Serbia; janaarsenijevic21@gmail.com; 10 Department of Surgery, Faculty of Medical Sciences, University of Kragujevac, 34000 Kragujevac, Serbia; danijeladrjovanovic@gmail.com; 11 Department of Pediatrics, Faculty of Medical Sciences, University of Kragujevac, 34000 Kragujevac, Serbia; sanjaknez1980@yahoo.com (S.K.); nevena.folic@yahoo.com (N.F.)

**Keywords:** adverse drug effect, schizophrenia, quality of life

## Abstract

**Background/Objectives**: Adverse effects of antipsychotics represent a significant limiting factor in achieving favorable therapeutic outcomes in the treatment of schizophrenia, and may contribute to a diminished quality of life among affected individuals. The primary objective of this study was to identify and evaluate the adverse effects of antipsychotics in patients diagnosed with schizophrenia who were treated at the social welfare institution, as well as to analyze the impact of these adverse effects on patients’ overall quality of life. **Methods**: A clinical, observational cross-sectional study was conducted, involving a sample of 278 patients diagnosed with schizophrenia. The patients were assessed in terms of their sociodemographic and clinical characteristics. Adverse effects of antipsychotics were evaluated using The Udvalg for Kliniske Undersøgelser (UKU) Side Effect Rating Scale, while quality of life was assessed in the previous study. **Results**: The average number of adverse effects per patient with schizophrenia was 3.56 for psychiatric, 1.18 for neurological, 2.62 for autonomic, and 7.12 for other side effects. The average UKU score was 17.22 ± 11.04, with significant differences based on accommodation, antipsychotic type, and dosing regimen. UKU scores were negatively correlated with the EuroQoL 5-Dimension 5-Level (EQ-5D-5L) index, Visual Analog Scale (VAS) score, the Quality-of-Life Enjoyment and Satisfaction Questionnaire—Short Form (Q-LES-Q-SF) score, and the scores of physical and psychological domains of the World Health Organization Quality-of-Life Scale (WHOQOL-BREF). **Conclusions**: The findings of this study suggest that the presence of antipsychotic-related adverse effects is a significant determinant that can negatively influence the quality of life in patients with schizophrenia. These results underscore the importance of an individualized approach when determining pharmacological treatment strategies in the management of schizophrenia.

## 1. Introduction

Schizophrenia (SCH) represents a mental illness of a chronic course and progressive dynamics that induces significant social and professional dysfunction and reduces the quality of life of patients [1,2]. The treatment of SCH requires a comprehensive and multimodal approach, with strategies commonly involving pharmacological measures and the dominant use of antipsychotics, along with non-pharmacological interventions aiming at achieving long-term positive treatment outcomes [3].

Antipsychotics utilized in the management of SCH exhibit a broad spectrum of efficacy encompassing the majority of the disease’s symptoms [4]. The use of these drugs necessitates an individualized approach regarding choosing one or more antipsychotics, the regimen of dosage (number and amount of daily dose), as well as systematic monitoring of therapeutic outcomes and adverse effects. While these pharmaceuticals contribute to the amelioration of various symptom clusters associated with the disorder, they concurrently pose risks for the emergence of diverse side effects and complications [5,6,7]. In terms of safety profile, first-generation antipsychotics are linked with a greater tendency to cause extrapyramidal side effects, while the use of second-generation antipsychotics is associated with a higher risk of so-called metabolic side effects, especially weight gain [8]. Consideration of the repercussions of antipsychotic-related side effects on the escalation of overall healthcare costs, the increase in cumulative mortality, and the resultant effects on the quality of life for individuals with SCH underscores the imperative for prudence in prescribing antipsychotics characterized by a heightened incidence of adverse reactions, in conjunction with adherence to pharmacovigilance practice [9,10].

Over the past two decades, the measurement of quality of life and analysis of factors influencing it have become an integral part of patient care [4]. Drug-related factors, such as adverse drug reactions, also play a significant role in determining the quality of life of these patients. The occurrence of adverse drug reactions can diminish the positive therapeutic outcomes of the administered therapy and contribute to reduced adherence and decreased quality of life of patients with SCH. Considering these facts, current therapeutic guidelines for the treatment of SCH incorporate strategies aimed at mitigating adverse drug reactions, emphasizing the importance of their early recognition and timely management as a crucial treatment objective [11,12].

The primary objective of this study was to evaluate the adverse effects of antipsychotics in patients with schizophrenia who were treated in a social welfare institution, while the secondary objectives of the study were focused on examining the relationship between the safety profile of antipsychotics and the impact of adverse effects of antipsychotics as a predictor of quality of life.

## 2. Results

The study included 287 patients with schizophrenia receiving care in a social welfare institution, of whom 162 (56.4%) were male and 125 (43.6%) were female. The mean age of the patients was 52.03 ± 10.522 years, with the youngest participant being 20 years old and the oldest 81 years old. Additional sociodemographic characteristics of the participants are presented in Table 1.

Patients were classified into five distinct categories based on the subtype of schizophrenia: the first group consisted of individuals with residual schizophrenia, comprising 16.4% (47) of the sample; the second group included individuals with paranoid schizophrenia, representing 21.6% (62); the third group consisted of individuals with hebephrenic schizophrenia, comprising 13.9% (40); the fourth group included individuals with undifferentiated schizophrenia, accounting for 24.7% (71); and the fifth group consisted of individuals with simple schizophrenia, comprising 23.3% (67). Other clinical characteristics of the patients have been described in detail in a previous study [13].

The UKU Side Effect Rating Scale was used to assess the presence and severity of drug-related side effects (NDs). The presence of psychiatric, neurological, autonomic, and other drug-related side effects was examined. An overall assessment of the impact of existing side effects was made based on the degree to which these side effects interfere with the daily life of the patients. The distribution of responses regarding the presence of psychiatric side effects is presented in Table 2.

The distribution of responses regarding the presence of neurological and autonomic side effects of drugs in patients is presented in Table 3 and Table 4.

The distribution of side effects that are classified as other drug-related side effects is presented in Table 5.

The average number of psychiatric adverse effects of the therapy was 3.56 ± 2.578, neurological 1.18 ± 1.219, autonomic 2.62 ± 1.843, while the average number of other adverse effects was 7.12 ± 2.869.

The average score on the UKU Side Effect Rating Scale was 17.2195 ± 11.035, with scores ranging from 0 to 49. Taking into account all sociodemographic and clinical characteristics of the patients, a statistically significant difference in UKU scores was observed based on the type of accommodation. Patients housed in the third pavilion had significantly higher UKU scores compared to patients in the first and second pavilions. A statistically significant difference in UKU scores was also observed in relation to the type of antipsychotic (AP) prescribed, with the highest scores recorded for patients using only aripiprazole, while the lowest scores were noted for patients on a combination of risperidone and haloperidol. Furthermore, patients significantly differed in UKU scores based on the daily dosing regimen of APs; those with a three-times-daily dosing regimen achieved significantly higher UKU scores compared to those receiving any AP once or twice a day. A positive correlation was found between the UKU score and the chlorpromazine equivalent, while a negative correlation was found between the UKU score and the length of institutional stay (Table 6).

As shown in Table 6, the Kruskal–Wallis test (χ^2^ = 29.637, *p* = 0.002) revealed a statistically significant difference in the number of side effects between drug groups. The lowest median UKU scores were observed in patients treated with risperidone/haloperidol (9.0), risperidone alone (12.0), and olanzapine (13.5). In contrast, the highest scores were found among patients receiving combination therapies, including olanzapine/haloperidol and clozapine/haloperidol (22.0), as well as aripiprazole (23.0).

The majority of patients, specifically 49.5% (142), reported experiencing mild adverse effects that did not significantly affect their daily functioning. Meanwhile, 31.4% (90) of patients stated that they did not experience any adverse effects, and 19.2% (55) reported that the adverse effects moderately affected their daily life. When examining the sociodemographic and clinical characteristics of the patients, a statistically significant difference was observed in the following categories: age, duration of institutional stay, chlorpromazine equivalent, and the number of psychiatric, neurological, autonomic, and other adverse effects, in relation to the perceived impact of existing adverse effects on patients’ everyday life. Additionally, both the type of accommodation and the number of prescribed antipsychotics showed a statistically significant association with the overall assessment of the impact of adverse effects. Patients who reported adverse effects of drugs that moderately affected their daily functioning were statistically significantly younger and had shorter institutional stays compared to patients who reported no or only mild adverse effects. On the other hand, patients with a moderate level of adverse effects impact had significantly higher chlorpromazine-equivalent doses and a greater number of psychiatric, neurological, autonomic, and other adverse effects in comparison to those without or with only mild adverse effects (Table 7).

In the majority of patients, specifically 59.2% (170), the presence of adverse effects resulted in more frequent clinical monitoring. In 7.0% (20) of cases, it was necessary to reduce the dose and/or discontinue the therapy. A total of 33.8% (97) of respondents reported that adverse effects had no impact on the course of their treatment.

A negative correlation was observed between the UKU scale and the EQ-5D-5L index (r = −0.458), the VAS score (r = −0.262), the Q-LES-Q-SF score (r = −0.284), as well as with the physical (r = −0.345) and psychological (r = −0.357) domains of the WHOQOL-BREF questionnaire. The correlation between the social domain (r = −0.110) and the environmental domain (r = −0.048) was not statistically significant.

## 3. Discussion

Drug-related factors—particularly adverse drug reactions—have emerged as critical determinants of intended therapeutic outcomes. In patients with SCH, the occurrence of adverse effects can significantly compromise the therapeutic benefits of antipsychotic treatment, reduce medication adherence, and ultimately impair quality of life [4]. In light of these considerations, the primary aim of this study was to evaluate the presence and variety of antipsychotic-induced adverse effects and to assess their impact on the quality of life among patients with schizophrenia residing in a social welfare institution.

The findings of this research indicated that the average score on The UKU Side Effect Rating Scale was 17.219 ± 11.035 (range: 0–49). All four categories of adverse effects described in the UKU questionnaire—psychiatric, neurological, autonomic, and other—were observed in the study population. It is also worth noting that a considerable number of patients reported no adverse effects. The most frequently reported side effects included prolonged sleep duration, drowsiness/sedation, weakness/fatigue/increased tiredness, and decreased sexual desire—results that are consistent with findings from similarly designed studies [14,15].

The results further demonstrated a positive correlation between UKU scores and antipsychotic dosage expressed as chlorpromazine equivalents, indicating that the number of adverse effects increased with higher dosages. In general, antipsychotics exhibit dose-dependent effects not only in terms of efficacy but also in terms of adverse effects, such as Parkinsonism, hyperprolactinemia, weight gain, and neurocognitive impairments [16]. Consequently, the treatment of schizophrenia necessitates individualized therapeutic planning, which, in addition to pharmacological management, should also incorporate psychosocial support interventions.

Regarding the perceived impact of adverse effects on patients’ daily lives, the findings show that patients who reported a moderate level of impact were prescribed significantly higher chlorpromazine-equivalent doses and experienced a greater number of psychiatric, neurological, autonomic, and other adverse effects compared to patients with no or mild adverse effects. These patients were also predominantly placed in the third pavilion of the institution.

Out of the five pavilions in the institution, patients included in the study were accommodated in the first, second, and third pavilions, based on their functional abilities, capacity for personal hygiene, and the level of medical care needed. A significantly higher UKU score was observed among patients residing in the third pavilion compared to those in the first and second. Patients in the third pavilion required the most intensive daily medical care, demonstrated greater functional impairment, and had reduced ability to carry out daily tasks, were less engaged in occupational and work-related activities, and showed more severe psychiatric symptoms. These findings align with previous studies that have highlighted the impact of institutional accommodation type on quality of life in patients with schizophrenia [13]. The dose-dependent nature of antipsychotic adverse effects, along with the challenges associated with treating more severe forms of schizophrenia—such as those observed in patients housed in the third pavilion—may explain the results obtained, which are consistent with findings from other authors [16,17,18,19].

In terms of the consequences of existing adverse effects, the most frequently reported consequence was the need for more frequent clinical monitoring, observed in 59.2% of patients. Although most adverse effects detected were of mild or moderate intensity, the necessity for frequent monitoring suggests that pharmacovigilance practices in the institution where this study was conducted align with current recommendations, particularly concerning the timely detection of adverse effects and the application of appropriate care and therapy [20].

The number of adverse drug effects—particularly psychiatric ones—among patients with SCH residing in a social welfare institution was shown to negatively affect all quality-of-life measures. Specifically, adverse effects correlated negatively with scores on the physical and psychological domains of the WHOQOL-BREF, the EQ-5D-5L index (including the VAS), and the Q-LES-Q-SF scale. These findings collectively underscore the detrimental effect of adverse effects on patients’ quality of life.

Assessing the quality of life (QoL) in institutionalized patients with schizophrenia is essential for identifying key predictors that contribute to either its enhancement or deterioration. Schizophrenia is a complex psychiatric disorder characterized by dysfunction across multiple domains of personality and functioning. As such, it necessitates a comprehensive, multimodal approach to treatment. A range of evidence-based interventions is available for individuals diagnosed with schizophrenia, encompassing pharmacological therapy, psychoeducation, family-based approaches, cognitive–behavioral therapy, and psychosocial rehabilitation (e.g., life skills training). Moreover, supportive services such as facilitated assisted living, supported housing, and supported employment represent critical components of integrated care and should be widely accessible to this population. Central to this framework is a recovery-oriented model of care, which emphasizes patient agency, shared decision-making, and active participation in treatment planning—not only for individuals with schizophrenia but also for their families and caregivers [21,22,23]. Pharmacological treatment should be integrated with psychosocial interventions in order to optimize therapeutic outcomes. It is essential to engage individuals living with schizophrenia in the decision-making process regarding pharmacological treatment, employing a supported decision-making approach that respects patient autonomy and preferences. According to the World Health Organization’s Essential Medicines List (EML), recommended antipsychotic medications for the treatment of psychotic disorders include the following: fluphenazine (with therapeutic alternatives such as haloperidol decanoate and zuclopenthixol decanoate), haloperidol, (alternative: chlorpromazine), haloperidol injection, olanzapine, paliperidone (alternative: risperidone injection), risperidone (alternatives: aripiprazole, olanzapine, paliperidone, quetiapine), and clozapine (listed under the complementary section of the EML). Individuals experiencing a first episode of psychosis typically respond favorably to antipsychotic medications. Therefore, the selection of a specific antipsychotic should primarily be guided by its side effect profile. For adults experiencing a first episode of psychosis (including schizophrenia) who have achieved remission, maintenance therapy with antipsychotic medication should be provided for a minimum duration of 7 to 12 months. This approach should involve a careful consideration of treatment efficacy, potential side effects, and the individual’s preferences [22].

While this study primarily focused on the impact of adverse drug effects on QoL, it is crucial to recognize that numerous other factors may significantly influence outcomes in this domain. These include illness-specific characteristics such as duration and severity of the disorder, presence of comorbid conditions, as well as treatment-related factors, including the use of polypharmacy, and broader environmental or institutional influences. The presence and severity of side effects may not be solely attributable to pharmacotherapy; rather, they may also reflect the chronicity or severity of the illness itself. Patients with more advanced or treatment-resistant forms of schizophrenia are often prescribed higher dosages or multiple medications, thereby increasing their vulnerability to side effects [3]. Although polypharmacy may be clinically justified in some contexts, it is frequently associated with heightened risks of adverse drug reactions, drug–drug interactions, cognitive impairment, and decreased adherence to treatment—all of which can negatively impact QoL [24]. In addition, comorbidities such as cardiovascular disease, diabetes, and depression are prevalent in this population and independently diminish QoL by compromising both physical and psychological well-being [25].

Beyond clinical symptoms and treatment side effects, institutional factors exert a substantial influence on patients’ overall quality of life. The quality of psychiatric care, availability of psychosocial support, degree of patient autonomy, quality of staff–patient interactions, and the nature of the physical environment are all critical. Extended hospitalization may also contribute to social withdrawal and limit opportunities for reintegration into the community, further exacerbating reductions in QoL [26].

While this study provides valuable insights into the factors influencing the QoL of individuals with schizophrenia, several limitations should be noted. Firstly, the cross-sectional design of this study prevents the establishment of causal relationships between the variables examined. To fully capture the dynamic interplay between illness characteristics, treatment-related factors, and environmental influences on QoL, future research employing a longitudinal design is essential. Long-term studies would allow for a deeper understanding of how the severity and duration of schizophrenia, as well as the presence of comorbidities, influence QoL outcomes over time. Moreover, such designs would enable the assessment of the long-term safety profiles of pharmacological treatments, as well as the effects of polypharmacy and institutional factors on patient well-being. Addressing these gaps will contribute to more robust and comprehensive strategies for improving the care and quality of life for individuals with schizophrenia.

## 4. Materials and Methods

This research involved conducting a clinical, observational, cross-sectional study, carried out in a social welfare institution in the central region of Serbia that provides care for patients from all over Serbia who suffer from long-term psychiatric disorders, including intellectual and mental difficulties that require round-the-clock supervision, care, and support that the family or community cannot adequately provide. This research focused on users of this institution who had a diagnosis of schizophrenia (F20.0–F20.9) based on the tenth revision of the International Classification of Diseases [27]. The time horizon for conducting the research was from 1 April 2023, to 1 December 2023. The competent Ethics Committee of the institution where research was performed and the Ministry of Labor and Social Policy, Sector for Family and Social Community Care, approved the ethical aspects of conducting this research (Ethical Committee Decision Number 01-1324/2).

Inclusion criteria encompassed the following characteristics: adult patients of both sexes with a verified diagnosis of schizophrenia with preserved work ability, length of stay and treatment in a social welfare institution of at least 12 months, sustained capacity of the respondent to make decisions and understand and follow the procedures used in the research, and signed voluntary consent to participate in the research. Exclusion criteria consisted of conditions such as dementia, autism, mental retardation, cognitive disorders that affect understanding and ability to respond to questionnaire questions, illiteracy of patients, and visual impairments disabled reading the questionnaire, as we reported in the previous study [13]. The total number of patients included in this survey was 278, which accounted for 153 patients from a previous pilot study conducted by the authors [28].

The design of this research included the collection of sociodemographic data and clinical characterizations of patients such as gender, age, education, presence of general comorbidities, and psychiatric comorbidities, patient habits (smoking status, coffee consumption), type of accommodation (first, second and third pavilion), type and duration of schizophrenia, length of stay in the institution, group (typical, atypical, combination) and type of antipsychotic, number of antipsychotic drugs prescribed per patient, antipsychotic dosage regimen, administered dose of antipsychotic (expressed in chlorpromazine equivalents), and number and type of adverse drug reactions, as it was presented in previous study [13]. These data were extracted from the patients’ medical records in accordance with daily obligations and patients’ needs, with the support of a multidisciplinary team of the institute comprising psychiatrists, speech therapists, social workers, occupational therapists, and medical technicians. Data collection was directed by a questionnaire developed specifically for the purpose of this study, based on the relevant literature addressing the quality of life in patients with SCH.

The methodological approach of estimating the quality of life of patients with schizophrenia who were treated in a social welfare institution was described in a previous study while assessing the presence and severity of side effects of antipsychotics was performed using a questionnaire entitled The Udvalg for Kliniske Undersøgelser Side Effect Rating Scale (The UKU-SERS) [13,29]. Permission was obtained from the authors and other copyright holders of all questionnaires used in this research. The assessment of individual adverse effects was conducted through interviews with each patient individually, following the instructions provided by the authors of the questionnaire. The interview was supplemented by clinical observation and information obtained from psychiatrists and medical staff at the institution, as well as by reviewing the patients’ medical records. The evaluation of a potential association between the administered drug and the occurrence of adverse drug reactions was grounded in clinical expertise and direct familiarity with the patients, as reported by psychiatrists within the institution who maintain daily contact with the patients and are responsible for ongoing monitoring and supervision of the prescribed therapy.

For the purposes of statistical analysis, IBM SPSS Statistics software, version 22 (IBM SPSS Statistics 22, Armonk, NY, USA), was utilized. Continuous variables are presented as means with standard deviations or as medians with interquartile ranges (IQR), as appropriate. Categorical variables are expressed as frequencies and percentages. The normality of data distribution was assessed using the Kolmogorov–Smirnov test. Comparisons of continuous variables between groups were conducted using the Mann–Whitney U test or the Kruskal–Wallis test, depending on the number of groups. Associations between categorical variables were analyzed using the chi-square test, while correlations between continuous variables were evaluated using either Pearson’s or Spearman’s correlation coefficients, depending on the distribution of the data.

## 5. Conclusions

This study highlights the significant impact of antipsychotic-induced adverse effects on the quality of life in institutionalized patients with schizophrenia. A clear dose-dependent relationship was observed, with higher chlorpromazine-equivalent doses associated with increased side effect burden and lower QoL scores. Patients with more severe functional impairment and greater care needs, particularly those in the third pavilion, experienced more pronounced adverse effects. These findings emphasize the importance of individualized treatment planning, routine pharmacovigilance, and the integration of psychosocial support to mitigate the negative effects of treatment and enhance patient well-being.

## Figures and Tables

**Table 1:** Sociodemographic characteristics of patients.

Sociodemographic Characteristics	N (%)
Education	
No formal primary education	24 (8.4)
Primary school	95 (33.1)
Secondary school	135 (47.0)
University degree	33 (11.5)
Caffeine consumption	
No	34 (11.8)
Yes	253 (88.2)
Alcohol consumption	
No	78 (27.2)
Yes	209 (72.8)
Type of accommodation	
First pavilion	130 (45.3)
Second pavilion	94 (32.8)
Third pavilion	63 (22.0)
Type of accommodation	
Four-bed rooms	12 (4.2)
Five-bed rooms	213 (74.3)
Six-bed rooms	62 (21.6)

**Table 2:** Distribution of responses regarding the presence of psychiatric side effects.

Psychiatric Side Effect		Not Present N (%)	Mild N (%)	Moderate N (%)	Severe N (%)	Not Assessed N (%)
1.1	Difficulty concentrating	152 (53.0)	71 (24.7)	54 (18.8)	10 (3.5)	-
1.2	Weakness/Fatigue/Increased tiredness	141 (49.1)	66 (23.0)	60 (20.9)	20 (7.0)	-
1.3	Drowsiness/Sedation	181 (63.1)	53 (18.5)	36 (12.5)	17 (5.9)	-
1.4	Impaired memory	164 (57.1)	65 (22.6)	46 (16.0)	12 (4.2)	-
1.5	Depression	154 (53.7)	37 (12.9)	45 (15.7)	51 (17.8)	-
1.6	Tension/Inner restlessness	157 (54.7)	52 (18.1)	46 (16.0)	32 (11.1)	-
1.7	Absence of emotion	231 (80.5)	30 (10.5)	19 (6.6)	7 (2.4)	-
1.8	Increased duration of sleep	238 (82.9)	14 (4.9)	15 (5.2)	20 (7.0)	-
1.9	Decreased duration of sleep	227 (79.1)	8 (2.8)	26 (9.1)	26 (9.1)	-
1.10	Increased activity during sleep	202 (70.4)	25 (8.7)	26 (9.1)	34 (11.8)	-

**Table 3:** Distribution of responses regarding the presence of neurological side effects.

Neurological Side Effect		Not Present N (%)	Mild N (%)	Moderate N (%)	Severe N (%)	Not Assessed N (%)
2.1	Distonia	257 (89.5)	25 (8.7)	5 (1.7)	-	-
2.2	Rigidity	261 (90.9)	22 (7.7)	4 (1.4)	-	-
2.3	Hypokinesia/Akinesia	258 (89.9)	22 (7.7)	5 (1.7)	2 (0.7)	-
2.4	Hyperkinesia	264 (92.0)	17 (5.9)	5 (1.7)	1 (0.3)	-
2.5	Tremor	171 (59.6)	47 (16.4)	46 (16.0)	23 (8.0)	-
2.6	Akathisia	259 (90.2)	22 (7.7)	5 (1.7)	1 (0.3)	-
2.7	Epileptic seizures	256 (89.2)	14 (4.9)	13 (4.5)	4 (1.4)	-
2.8	Paresthesia	230 (80.1)	38 (13.2)	15 (5.2)	4 (1.4)	-

**Table 4:** Distribution of responses regarding the presence of autonomic side effects.

Autonomic Side Effects		Not Present N (%)	Mild N (%)	Moderate N (%)	Severe N (%)	Not Assessed N (%)
3.1	Visual disturbances	224 (78.0)	35 (12.2)	17 (5.9)	11 (3.8)	-
3.2	Increased salivation	210 (73.2)	21 (7.3)	29 (10.1)	27 (9.4)	-
3.3	Decreased salivation	225 (78.4)	25 (8.7)	26 (9.1)	11 (3.8)	-
3.4	Nausea/Vomiting	245 (85.4)	32 (11.1)	7 (2.4)	3 (1.0)	-
3.5	Diarrhea	253 (88.2)	24 (8.4)	8 (2.8)	2 (0.7)	-
3.6	Constipation	247 (86.1)	24 (8.4)	10 (3.5)	6 (2.1)	-
3.7	Urinary disorders	193 (67.2)	31 (10.8)	37 (12.9)	26 (9.1)	-
3.8	Polyuria/Polydipsia	209 (72.8)	29 (10.1)	35 (12.2)	14 (4.9)	-
3.9	Dizziness	225 (78.4)	43 (15.0)	15 (5.2)	4 (1.4)	-
3.10	Palpitations/Tachycardia	208 (72.5)	54 (18.8)	22 (7.7)	3 (1.0)	-
3.11	Excessive sweating	166 (57.8)	53 (18.5)	46 (16.0)	22 (7.7)	-

**Table 5:** Distribution of responses regarding the presence of other side effects.

Other Side Effects		Not Present N (%)	Mild N (%)	Moderate N (%)	Severe N (%)	Not Assessed N (%)
4.1	Rash	282 (98.3)	3 (1.0)	1 (0.3)	1 (0.3)	-
4.1a	Morbilliform rash	287 (100)	-	-	-	-
4.1b	Petechiae	286 (99.7)	1 (0.3)	-	-	-
4.1c	Urticaria	284 (99.0)	1 (0.3)	2 (0.7)	-	-
4.1d	Psoriasis	280 (97.6)	6 (2.1)	1 (0.3)	-	-
4.1e	Cannot be classified	287 (100)	-	-	-	-
4.2	Pruritus	287 (100)	-	-	-	-
4.3	Photosensitivity	274 (95.5)	5 (1.7)	7 (2.4)	1 (0.3)	-
4.4	Hyperpigmentation	279 (97.2)	2 (0.7)	6 (2.1)	-	-
4.5	Weight gain	191 (66.6)	41 (14.3)	45 (15.7)	10 (3.5)	-
4.6	Wight loss	256 (89.2)	15 (5.2)	13 (4.5)	3 (1.0)	-
4.7	Menorrhagia	279 (97.2)	5 (1.7)	2 (0.7)	1 (0.3)	-
4.8	Amenorrhea	275 (95.8)	5 (1.7)	4 (1.4)	3 (1.0)	-
4.9	Galactorrhea	284 (99.0)	2 (0.7)	1 (0.3)	-	-
4.10	Gynecomastia	287 (100)	-	-	-	-
4.11	Increased sexual desire	233 (81.2)	5 (1.7)	28 (9.8)	21 (7.3)	-
4.12	Decreased sexual desire	186 (64.8)	10 (3.5)	60 (20.9)	31 (10.8)	-
4.13	Erectile dysfunction	221 (77.0)	14 (4.9)	41 (14.3)	11 (3.8)	-
4.14	Ejaculatory dysfunction	212 (73.9)	20 (7.0)	41 (14.3)	14 (4.9)	-
4.15	Orgasmic disorders	244 (85.0)	7 (2.4)	26 (9.1)	10 (3.5)	-
4.16	Vaginal dryness	247 (86.1)	12 (4.2)	24 (8.4)	4 (1.4)	-
4.17	Headache	201 (70.0)	43 (15.0)	39 (13.6)	4 (1.4)	-
4.17a	Tension headache	231 (80.5)	32 (11.1)	21 (7.3)	3 (1.0)	-
4.17b	Mygrene	278 (96.9)	6 (2.1)	3 (1.0)	-	-
4.17c	Others	286 (99.7)	1 (0.3)	-	-	-

**Table 6:** UKU score values in relation to patient characteristics.

Patient Characteristics	*UKU* Scale Median (IQR)	Kruskal–Wallis/*p*
**Type of accommodation**		
First pavilion	12.5 (16.25)	7.255/0.027
Second pavilion	15.0 (13.0)	
Third pavilion	20.0 (20.0)	
**Type of prescribed antipsychotics**		
Risperidone/Haloperidol	9.0 (9.25)	29.637/0.002
Risperidone	12.0 (12.5)	
Clozapine	13.5 (17.0)	
Olanzapine	13.0 (12.0)	
Haloperidol	13.0 (14.0)	
Risperidone/Olanzapine	18.5 (12.5)	
Clozapine/Olanzapine	13.5 (25.5)	
Clozapine/Olanzapine/Haloperidol	18.0 (17.0)	
Olanzapine/Haloperidol	22.0 (28.0)	
Clozapine/Haloperidol	22.0 (19.75)	
Risperidone/Clozapine	18.5 (15.5)	
Aripiprazole	23.0 (16.75)	
**Daily dosing schedule**		
Single dose per 24 h	13.5 (15.0)	9.692/0.008
Two doses per 24 h	13.0 (16.0)	
Three doses per 24 h	17.0 (16.75)	
	**Pearson r**	***p***
Length of institutional stay	−0.164	0.045
Chlorpromazine equivalent	0.243	0.000

**Table 7:** Values of age, duration of institutional stay, chlorpromazine equivalent, and number of psychiatric, neurological, autonomic, and other adverse effects in relation to the perceived impact of existing adverse effects on patients’ daily functioning.

	General Assessment of the Impact of Existing Adverse Effects on Daily Functioning			
	None Median (*IQR*)	Mild Median (*IQR*)	Moderate Median (*IQR*)	Kruskal Wallis/*p*
Age	56.0 (15.0)	53.0 (13.0)	49.0 (17.0)	7.231/0.027
Duration of institutional stay	13.0 (17.0)	9.0 (14.0)	6.0 (13.0)	9.039/0.011
Chlorpromazine equivalent	287.5 (250.0)	318.75 (315.63)	400.0 (425.0)	21.084/0.000
Number of psychiatric adverse effects	2.0 (3.0)	3.0 (3.0)	6.0 (4.0)	55.015/0.000
Number of neurological adverse effects	0.0 (1.0)	1.0 (2.0)	2.0 (2.0)	46.366/0.000
Number of autonomic adverse effects	1.0 (3.0)	3.0 (3.0)	4.0 (2.0)	51.652/0.000
Number of other adverse effects	5.0 (4.0)	7.0 (4.0)	10.0 (3.0)	85.537/0.00
Type of accommodation				**Chi-square/*p***
First pavilion	55 (61.1)	57 (40.1)	18 (32.7)	16.471/0.002
Second pavilion	20 (22.2)	55 (38.7)	19 (34.5)	
Third pavilion	15 (16.7)	30 (21.1)	18 (32.7)	
Number of prescribed antipsychotics				
One	60 (66.7)	89 (62.7)	25 (45.5)	12.617/0.013
Two	26 (28.9)	48 (33.8)	22 (40.0)	
Three	4 (4.4)	5 (3.5)	8 (14.5)	

## Data Availability

The data presented in this study are available upon request.

## Abbreviations

The following abbreviations are used in this manuscript:

SCH	Schizophrenia
AE or SE or ADR	Adverse Effect or Side Effect or Adverse Drug Reactions
The UKU-SERS	The Udvalg for Kliniske Undersøgelser Side Effect Rating Scale
EQ-5D-5L	EuroQoL 5-Dimension 5-Level
VAS	Visual Analog Scale
The Q-LES-Q-SF	The Quality-of-Life Enjoyment and Satisfaction Questionnaire—Short Form
WHOQOL-BREF	The World Health Organization Quality-of-Life Scale
IQR	Interquartile range
QoL	Quality of life
EML	Essential Medicines List
